# Oral Administration of *Trichosanthes Kirilowii* Fruit Extract Ameliorates Airway Inflammation and Suppresses Th2 Cell Activities in Ovalbumin‐Sensitized Mice

**DOI:** 10.1002/kjm2.70055

**Published:** 2025-05-29

**Authors:** Ting‐Chi Lin, Wen‐Chung Huang, Chwan‐Fwu Lin, Wan‐Ting Chi, Shu‐Chen Cheng, Nai‐Chun Ting, Chian‐Jiun Liou

**Affiliations:** ^1^ Department of Traditional Chinese Medicine Chang Gung Memorial Hospital Taoyuan City Taiwan; ^2^ Graduate Institute of Health Industry Technology, Center for Drug Research and Development Chang Gung University of Science and Technology Taoyuan City Taiwan; ^3^ Department of Cosmetic Science, Center for Drug Research and Development Chang Gung University of Science and Technology Taoyuan City Taiwan; ^4^ Department of Anesthesiology Chang Gung Memorial Hospital Taoyuan City Taiwan; ^5^ Division of Pulmonary, Allergy and Critical Care Medicine University of Pittsburgh School of Medicine Pittsburgh Pennsylvania USA; ^6^ Department of Nursing, Division of Basic Medical Sciences, Center for Drug Research and Development Chang Gung University of Science and Technology Taoyuan City Taiwan

**Keywords:** airway hyperresponsiveness, airway inflammation, asthma, eosinophil, *Trichosanthes kirilowii* fruit extract

## Abstract

*Trichosanthes kirilowii* is a plant used in traditional Chinese medicine. Its fruits, seeds, and roots can all be used medicinally. The fruit of *T. kirilowii* has a moistening effect on the lung for arresting cough. However, there is no scientific research to prove that the fruit of *T. kirilowii* can improve asthma. Herein, we evaluated whether *T. kirilowii* fruit extract (TK) could reduce airway inflammation and airway hyperresponsiveness (AHR) in a murine model of asthma. Female BALB/c mice were sensitized with ovalbumin to induce asthma and treated with varying oral doses of TK from days 14 to 27 of the experiment. Additionally, IL‐4/TNF‐α‐stimulated BEAS‐2B cells were treated with different doses of TK to investigate inflammatory cytokine and chemokine secretion. TK treatment significantly mitigated AHR, eosinophil infiltration, and airway inflammation in the lungs of asthmatic mice. It also alleviated goblet cell hyperplasia and lung ROS levels while modulating Th2‐associated cytokine levels in the bronchoalveolar lavage fluid. Moreover, TK effectively alleviated proinflammatory cytokine, chemokine, and eotaxin in IL‐4 and TNF‐α‐stimulated BEAS‐2B cells. Our results indicate that TK effectively improved asthma symptoms in mice through suppressed Th2‐cell activation, which mitigated airway inflammation, oxidative stress, and AHR.

## 1 Introduction

Asthma is a persistent condition characterized by inflammation and hypersensitivity of airways, leading to difficulties in breathing. In the patient with asthma, inhaled allergens and air pollutants can induce repeated airway contractions, excessive mucus secretion by the trachea and bronchi, and wheezing and dyspnea [1]. Its global prevalence is rising and closely related to the expansion of allergen distribution caused by climate warming and the severe deterioration of air quality [2]. Allergic asthma, the most common form, is characterized by eosinophilic lung infiltration, airway inflammation, and oxidative stress, leading to airway hyperresponsiveness (AHR) and elevated serum IgE levels [3].

The development of allergic asthma is strongly associated with overactivation of Th2 cells [4]. Allergens stimulate Th2 cell activation and release excessive Th2 cytokines [5]. IL‐4 promotes B cells to activate and differentiate into plasma cells; subsequent antibody‐class switching from IgM to IgE results in excess IgE in the serum and lungs of asthma patients. IL‐5 promotes the differentiation of bone marrow cells into eosinophils, which migrate to the lungs under the influence of eotaxins [4]. In addition, copious secretion of IL‐13 worsens AHR and increases the proliferation of mucus in tracheal epithelial cells [3]. Therefore, inhibiting the excessive activation of Th2 cells can improve asthma pathology.

Clinical medications for asthma management and prevention are primarily categorized into two types [6]. The first type focuses on controlling and preventing asthma attacks by alleviating airway inflammation and reducing airway remodeling. Commonly used ones include oral and inhaled steroids and long‐acting β2 agonists [7]. The second type of asthma drugs is bronchodilators, which are mainly used to relieve dyspnea caused by airway constriction. Commonly used ones include short‐acting β2 agonists and anticholinergics [6]. In East Asia, traditional Chinese medicine is a widely used alternative approach for treating and improving asthma symptoms. Herbal formulas such as Ding Chuan Tang and Xiao‐Qing‐Long‐Tang are commonly employed to manage asthma symptoms [8].

*Trichosanthes kirilowii* is a perennial cucurbitaceous plant widely distributed in East Asia. In traditional Chinese medicine, *T. kirilowii* fruit is used to treat excessive phlegm and relieve coughs [9]. In a Taiwanese study, the traditional Chinese medicine formula NRICM101 (containing *T. kirilowii* fruit) was reported to slow the inflammation of acute lung injury in COVID‐19 patients [10]. However, whether *T. kirilowii* fruit extract (TK) inhibits the Th2 response and airway inflammation in asthmatic mice remains unclear. Herein, we investigate whether TK can alleviate airway inflammation and oxidative stress while modulating Th2‐related cytokine expression in asthmatic mice.

## 2 Materials and Methods

### 2.1 Preparation of *T. kirilowii* Fruit Extracts

The preparation of TK was conducted by the Department of Chinese Herbal Pharmacy at Chang Gung Memorial Hospital (Taoyuan, Taiwan). In summary, 500 g of the fruit were processed and dried in accordance with traditional Chinese herbal practices. The dried fruit was then soaked in water and boiled for 1 h, resulting in a crude extract that was subsequently filtered and lyophilized, yielding 42.2 g of TK, which equates to an 8.44% yield (w/w). The TK solution underwent overnight shaking, followed by filtration through a 0.45‐nm filter for sterilization, and was stored at −80°C.

### 2.2 High‐Performance Liquid Chromatography (HPLC) Analysis

TK solution filtered through a 0.45‐μm PVDF filter before injection into an HPLC system (Shimadzu Nexera‐I LC‐2040C‐3D, Kyoto, Japan) for fingerprint analysis. A standard sample of 3,29‐dibenzoyl karounitriol (DK), dissolved in methanol, was used for comparison. The separation process utilized a reverse‐phase column (Cosmosil 5C18‐AR‐II, 5 μm, 4.6 mm × 250 mm) at a flow rate of 1.0 mL/min with a gradient mobile phase composed of A (H_2_O) and B (acetonitrile):0–5 min, 50%–0% A and 50%–100% B; 5–40 min, 0% A and 100% B. The injection volume was 20 μL, the column temperature was 35°C, and UV detection was conducted at 230 nm.

### 2.3 Mice

In terms of animal studies, female BALB/c mice aged between 6 to 8 weeks were obtained from the National Laboratory Animal Center in Taipei, Taiwan. They were kept under standard conditions with a controlled light/dark cycle. All experimental procedures adhered to protocols approved by the Laboratory Animal Care Committee at Chang Gung University of Science and Technology (IACUC approval number: 2023–003).

### 2.4 Mouse Sensitization and TK Treatment

For mouse sensitization and treatment with TK, 40 mice were randomly assigned into four groups (*n* = 10 each): (1) normal control mice (N group); (2) OVA‐sensitized mice (OVA group); (3) OVA‐sensitized mice treated with TK at a dosage of 100 mg/kg (TK100 group) or 200 mg/kg (TK200 group). Sensitization involved intraperitoneal injections of 50 μg OVA (grade V; Sigma, St. Louis, MO, USA) mixed with adjuvant (Al (OH)_3_) (Thermo Fisher Scientific, Rockford, IL, USA) on days 1–3 and day 14 [11]. Mice were then exposed to a 2% OVA solution for 30 min on days 14, 17, 20, 23, and 27 using a nebulizer (DeVilbiss Pulmo‐Aide 5650D, USA). The atomized particle size ranged from 0.5 to 5 μm and the nebulization rate was 0.15–0.35 mL/min. From days fourteen to twenty‐seven, saline was administered to the *N* and OVA groups, and 100 or 200 mg/kg TK was fed orally with a gastric feeding tube daily to the TK100 and TK200 groups, respectively.

### 2.5 AHR Measurement

On day 28, the mice underwent exposure to escalating concentrations of methacholine (0–40 mg/mL) through inhalation over a period of 3 min. Following this exposure, they were placed in a whole‐body plethysmography chamber (Buxco Electronics, Troy, NY, USA) to measure airway hyperresponsiveness (AHR), recorded as enhanced pause (Penh) values [12].

### 2.6 Bronchoalveolar Lavage Fluid

For bronchoalveolar lavage fluid (BALF) collection, the mice were anesthetized, and BALF was obtained by inserting a needle into the trachea [11]. The collected fluid was then centrifuged, allowing for the supernatant to be analyzed for cytokines and chemokines. Next, cells were stained with Giemsa solution (Sigma) to evaluate the total cell count and identify different types of immune cells.

### 2.7 Histologic Analysis of Lung

In histological examination of lung tissue, samples were fixed in formalin, embedded in paraffin, and sectioned into 6‐μm slices. Hematoxylin and eosin (HE) staining was performed to assess eosinophil infiltration, which was graded using a five‐point pathology scale [13]. Periodic acid‐Schiff (PAS; Sigma) staining investigated goblet cell hyperplasia in the trachea, while Masson's trichrome (Sigma) staining observed collagen distribution within lung tissue. Immunohistochemical staining was detected.

NF‐κB expression in lung tissue. Briefly, lung sections were treated with p65 antibody (Cell Signaling Technology, MA, USA) overnight. The slides were then treated with secondary antibody and DAB substrate solution to detect p65 expression [11].

### 2.8 Oxidative Damage–Related Enzymes of Lung

To analyze oxidative damage‐related enzymes in lung tissues, samples were homogenized using a homogenizer (MP Biomedicals, Santa Ana, CA, USA). The resulting extract was centrifuged to obtain the supernatant for measuring levels of malondialdehyde (MDA), glutathione (GSH), catalase (CAT), and superoxide dismutase (SOD) (Sigma), following the protocols provided with the detection reagents [12].

### 2.9 Serum Collection and Splenocyte Cultures

For serum collection and splenocyte cultures, mouse serum was gathered and stored at −80°C for subsequent analysis. OVA‐specific antibodies present in the serum were quantified using ELISA. Single splenocytes were isolated and treated with 100 μg/mL OVA for 5 days, after which cytokine levels in the culture supernatant were analyzed via ELISA.

### 2.10 Biochemical Analysis of Serum

Serum glutamic pyruvic transaminase (GPT) and glutamate oxaloacetate transaminase (GOT) levels were determined using a DRI‐CHEM NX500 biochemical analyzer (Fujifilm Co., Tokyo, Japan).

### 2.11 Cell Viability Assay

The human bronchial epithelial cell line BEAS‐2B, sourced from the American Type Culture Collection (Manassas, VA, USA), was used to evaluate cell viability via the CCK‐8 assay (Sigma). Cells were exposed to various concentrations of TK (0–200 μg/mL) for 24 h, followed by incubation with CCK‐8 reagent for 4 h. Absorbance at 450 nm was measured to assess viability.

### 2.12 TK Treatment of BEAS‐2B Cells

BEAS‐2B cells pre‐incubated with TK (0–100 μg/mL) for 1 h prior to stimulation with IL‐4 and TNF‐α (10 ng/mL) for 24 h. Levels of cytokines and chemokines in the culture supernatant were measured using ELISA.

### 2.13 ELISA

OVA‐specific antibodies in serum were measured with ELISA kits from BD Biosciences (San Diego, CA, USA). Cytokine and chemokine concentrations were assessed using R&D Systems ELISA kits (Minneapolis, MN, USA) and analyzed via a microplate reader (Thermo Fisher).

### 2.14 Reactive Oxygen Species Assay

TK treated BEAS‐2B cells stimulated with IL‐4/TNF‐α (10 ng/mL), and incubated with 20 μM DCFH‐DA for 30 min. ROS levels were determined by observing fluorescence intensity under a microscope (Olympus, Tokyo, Japan).

### 2.15 Western Blots

Proteins were separated by SDS polyacrylamide gel electrophoresis. The proteins were transferred to PVDF membrane, which was incubated with specific antibodies. Then, the membranes were incubated with secondary antibodies, and proteins were detected by Biospectrum Imaging System (UVP, Upland, CA, USA). Specific antibodies included Phospho‐NF‐κB p65, NF‐κB p65 (Cell Signaling Technology), and β‐actin (Sigma).

### 2.16 Statistical Methods

Results are expressed as mean ± SEM from a minimum of three independent experiments. Statistical significance was evaluated using one‐way ANOVA followed by Dunnett's post hoc test, with a threshold of *p* < 0.05.

## 3 Results

### 3.1 TK Improved AHR and Reduced Inflammatory Cells in the BALF

**FIGURE 1:**
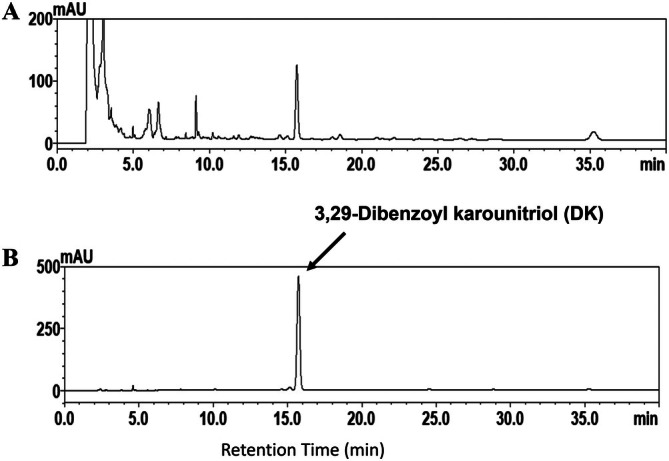
HPLC profiles of *Trichosanthes kirilowii* (TK) extracts. (A) HPLC fingerprint of TK extract; the major peaks on the TK profile were identified using 3,29‐dibenzoyl karounitriol (DK) as a standard. (B) HPLC fingerprint of the DK standard.

**FIGURE 2:**
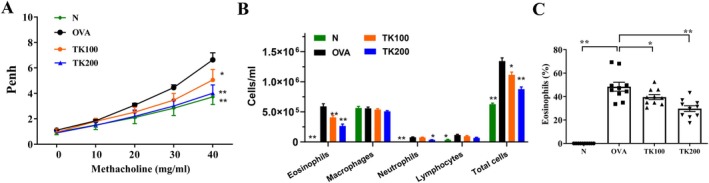
Effects of *T. kirilowii* fruit extract (TK) on airway hyperresponsiveness (AHR) and cell counts in bronchoalveolar lavage fluid (BALF) of asthmatic mice. (A) Mice inhaled increasing doses of methacholine, and AHR was assessed (shown as Penh values). (B) The inflammatory cells in BALF were counted, including total cells, eosinophils, neutrophils, lymphocytes, and macrophages. (C) Percentage of eosinophils in BALF. Three independent experiments were analyzed. Data are presented as mean ± SEM. *p < 0.05, **p < 0.01 compared with the OVA control group.

The HPLC fingerprint analysis of TK revealed that DK eluted at 16.4 min, serving as a distinct marker for the identification of *T. kirilowii* (Figure 1). OVA‐sensitized mice displayed significantly elevated Penh values, indicative of heightened AHR, compared to normal controls. At 40 mg/mL methacholine, TK100 and TK200 treatments significantly lowered Penh values (Figure 2A). OVA‐sensitized asthmatic mice treated with TK could significantly reduce the numbers of eosinophils (TK100: 4.08 × 10^5^ ± 3.13 × 10^4^, *p* < 0.01; TK200: 2.66 × 10^5^ ± 9.84 × 10^4^, *p* < 0.01), compared to the OVA group (5.91 × 10^5^ ± 4.49 × 10^4^; Figure 2B). We also found that the total cell numbers in asthmatic mice were significantly reduced after treatment with TK (TK100: 1.12 × 10^6^ ± 4.65 × 10^4^, *p* < 0.05; TK200: 8.78 × 10^5^ ± 3.69 × 10^4^, *p* < 0.01) compared to the OVA group (1.34 × 10^6^ ± 5.27 × 10^4^; Figure 2B). TK treatment did not significantly regulate macrophage and lymphocyte counts in asthmatic mice. Furthermore, the numbers of neutrophils in asthmatic mice were significantly reduced after treatment with TK200 (Figure 2B). Asthmatic mice treated with TK had a significantly reduced proportion of eosinophils compared to the OVA group (TK100: 39.5% ± 2.1%, *p* < 0.05; TK200: 29.7% ± 2.4%, *p* < 0.01 vs. OVA: 48.4% ± 3.7%) (Figure 2C). Our results showed that TK can effectively reduce AHR and the number of inflammatory immune cells in the BALF.

### 3.2 TK Inhibited Eosinophil Infiltration and Goblet Cell Hyperplasia in the Lung

**FIGURE 3:**
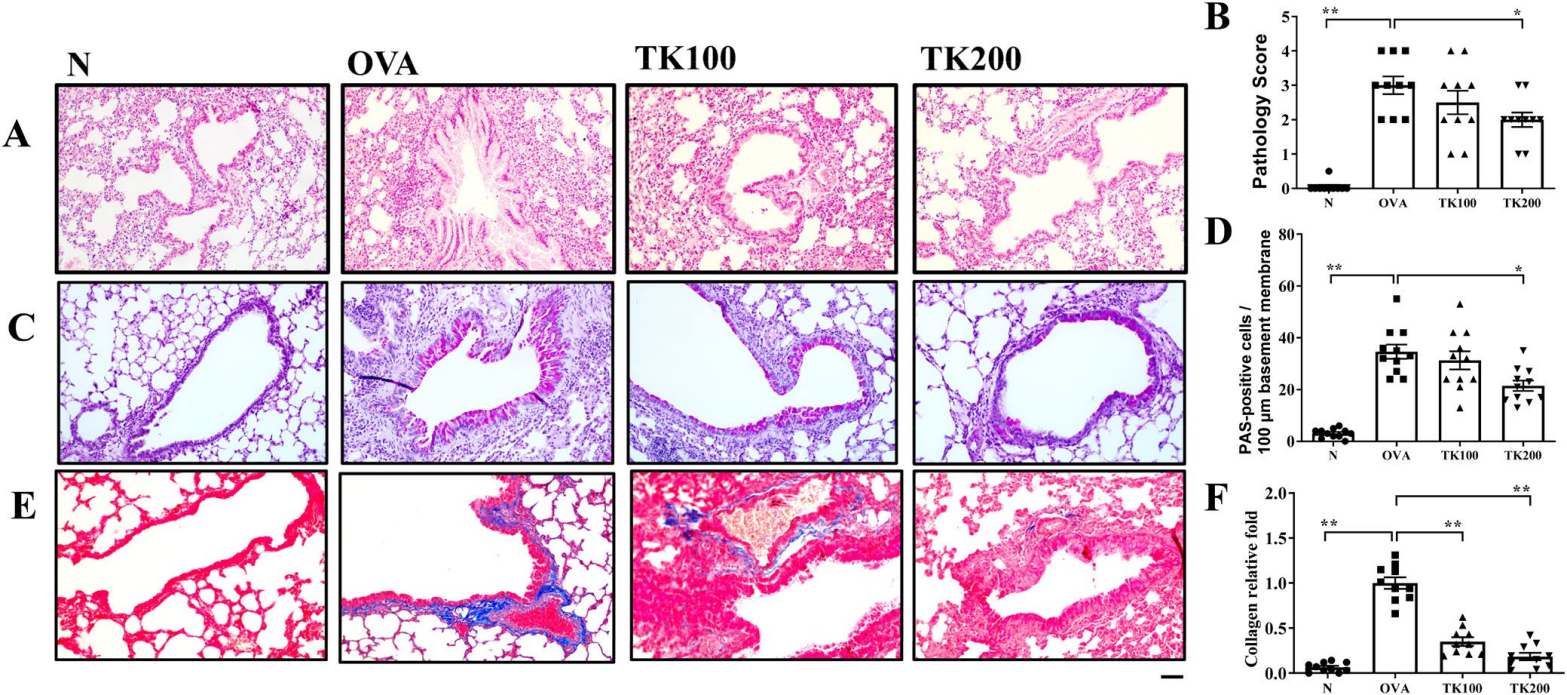
*T. kirilowii* fruit extract (TK) effects on asthmatic lung tissue. TK reduced (A) eosinophil infiltration (HE stain; 200× magnification) and (B) the inflammatory score. (C) PAS‐stained lung sections displaying goblet cell hyperplasia (200× magnification). (D) The number of PAS‐positive cells per 100 μm of basement membrane. Lung sections were stained with (E) Masson trichrome stain to detect collagen expression (200× magnification) for (F) quantitative analysis of collagen. Values are the mean ± SEM. *p < 0.05, **p < 0.01 versus the OVA group (scale bar = 100 μm).

**FIGURE 4:**
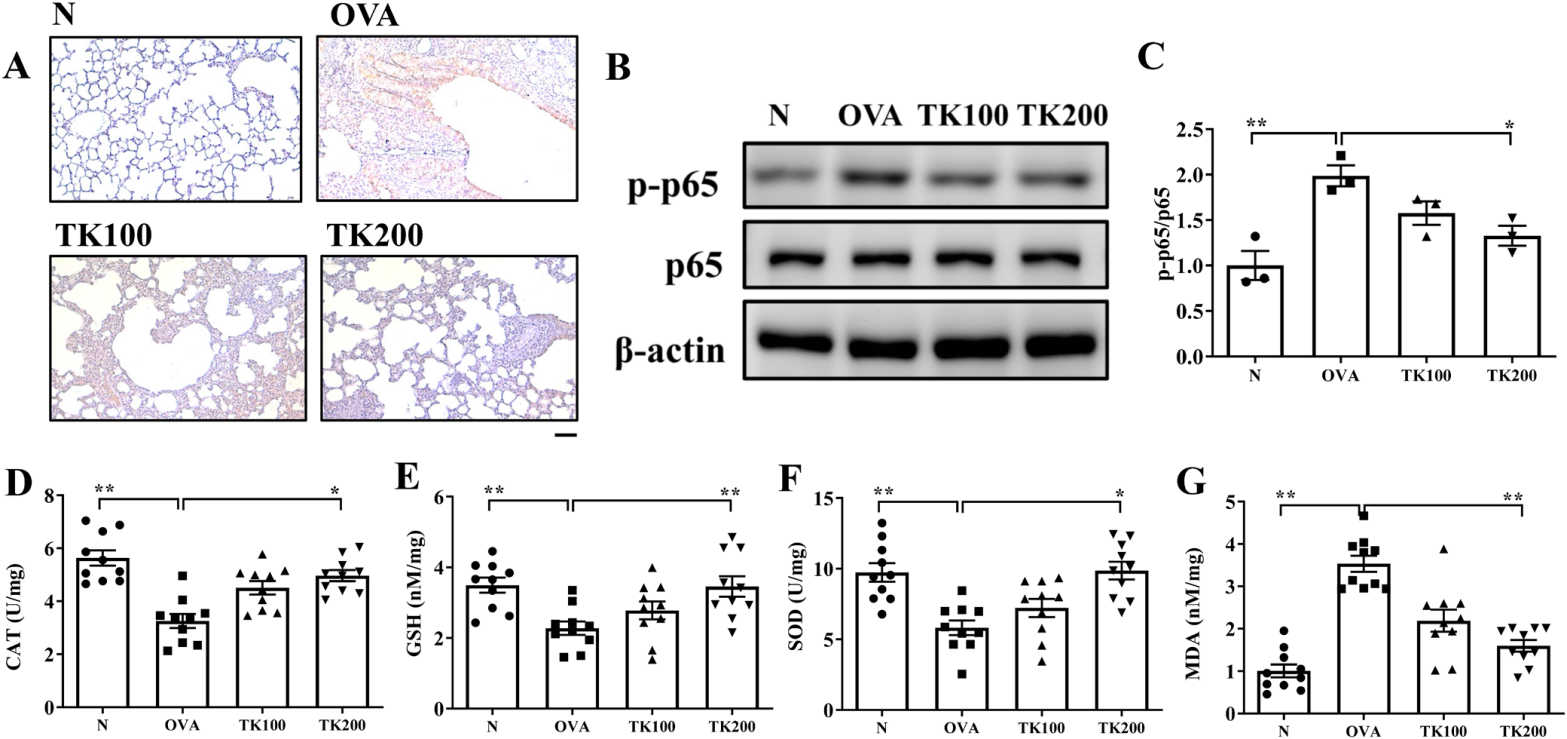
*T. kirilowii* fruit extract (TK) effects on the phosphorylation of NF‐κB and oxidative stress factors. (A) Immunohistochemistry stain detected NF‐κB expression in lung tissue(scale bar = 100 μm). (B) The phosphorylation of p65 (p‐p65, NF‐κB subunit) expression in lung tissue, and (C) fold‐change relative to p‐p65/p65 expression. (D) Catalase (CAT), (E) glutathione (GSH), (F) superoxide dismutase (SOD), and (G) malondialdehyde (MDA) activities in mouse lung tissues. Values are the mean ± SEM. *p < 0.05, **p < 0.01 versus the OVA group.

OVA‐sensitized mice demonstrated a significant increase in eosinophil infiltration relative to the normal control group. Treatment with TK notably decreased eosinophil levels and improved the inflammatory pathology scores in the lungs when compared to the untreated OVA group. (Figure 3A,B). Additionally, periodic acid‐Schiff (PAS) staining showed that mice treated with TK had a marked reduction in hyperplasia of tracheal goblet cells compared to those in the OVA group (Figure 3C,D). Masson's trichrome staining further revealed that collagen production in the lungs was significantly lower in TK‐treated OVA‐sensitized mice than in the OVA group (Figure 3E,F). Immunohistochemical staining demonstrated that TK suppressed NF‐κB production in the lungs of asthmatic mice (Figure 4A). Next, OVA‐sensitized asthmatic mice treated with TK200 could significantly suppress the phosphorylation of p65 (NF‐κB subunit), compared to the OVA group (Figure 4B,C).

### 3.3 TK Regulated Antioxidant Enzyme Expression in Lung

In lung tissue, OVA‐sensitized mice exhibited significantly elevated levels of MDA alongside decreased production of GSH, CAT, and SOD when compared to normal mice. Treatment with TK at a dosage of 200 mg/kg notably increased GSH, CAT, and SOD levels while simultaneously lowering MDA levels in the OVA‐sensitized group (Figure 4D–G).

### 3.4 TK Regulated Cytokine and Chemokine Levels in BALF

**FIGURE 5:**
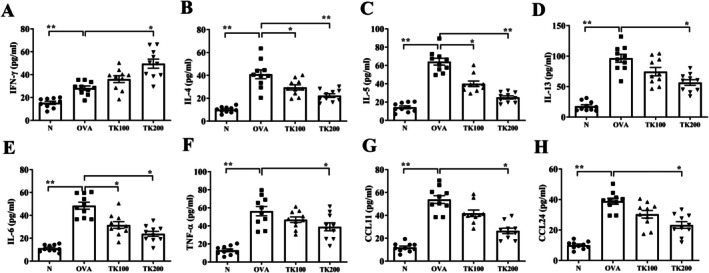
Effects of *T. kirilowii* fruit extract (TK) on the levels of cytokines and chemokines in bronchoalveolar lavage fluid (BALF). The concentrations of (A) IFN‐γ, (B) IL‐4, (C) IL‐5, (D) IL‐13, (E) IL‐6, (F) TNF‐α, (G) CCL11, and (H) CCL24 were measured in BALF. Three independent experiments were analyzed. All data are presented as mean ± SEM. *p < 0.05, **p < 0.01 compared with the OVA control group.

With respect to the regulation of cytokine and chemokine levels in BALF, asthmatic mice receiving TK treatment showed enhanced levels of IFN‐γ and reduced concentrations of IL‐4, IL‐5, IL‐13, TNF‐α, IL‐6, CCL11, and CCL24 compared to untreated OVA‐sensitized mice (Figure 5).

### 3.5 TK Regulates Antibody and Cytokine Levels in Serum and Splenocytes

**FIGURE 6:**
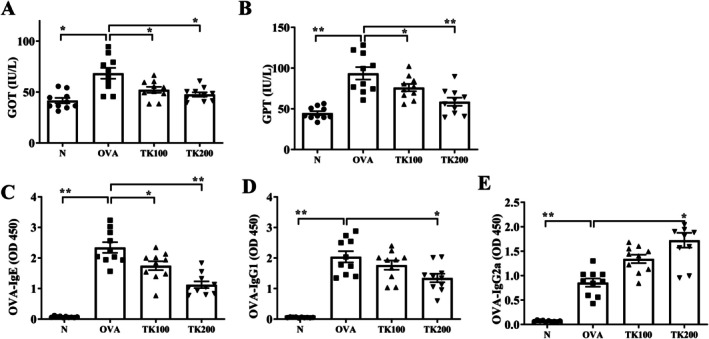
Effects of *T. kirilowii* fruit extract (TK) on liver enzymes and OVA‐specific antibodies in serum. Serum levels of (A) GOT, (B) GPT, (C) OVA‐IgE, (D) OVA‐IgG1, and (E) OVA‐IgG2a from mice without or with TK treatment. Three independent experiments were analyzed. All data are presented as mean ± SEM. *p < 0.05, **p < 0.01 compared with the OVA control group.

**FIGURE 7:**
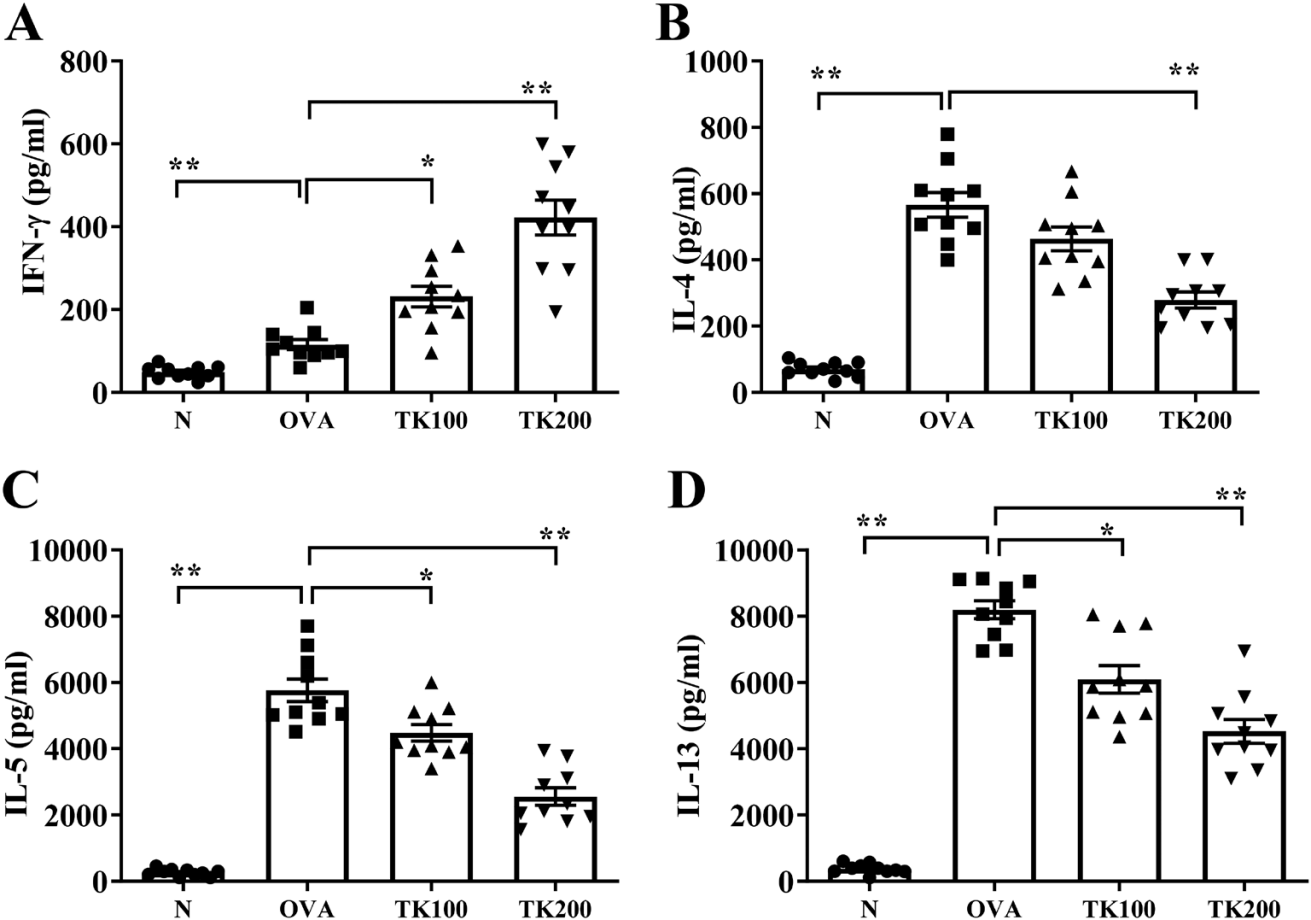
Effects of *T. kirilowii* fruit extract (TK) on cytokine production in OVA‐activated spleen cells. (A) IFN‐γ, (B) IL‐4, (C) IL‐5, and (D) IL‐13 levels were measured by ELISA. Three independent experiments were analyzed. All data are presented as mean ± SEM. *p < 0.05, **p < 0.01 compared with the OVA control group.

We measured serum GOT and GPT levels to evaluate whether administration of TK would cause liver and kidney toxicity [14]. The result demonstrated that TK‐treated asthmatic mice had lower serum levels of GOT and GPT (Figure 6A,B). Furthermore, Th1 cells can stimulate B cells to secrete IgG2a. Th2 cells can induce B cells to secrete antibodies, such as IgG1 and IgE. Therefore, IgG1 and IgG2a expressions can be used as indicators of Th1 cell and Th2 cell activation [15, 16]. TK‐treated asthmatic mice also exhibited reduced levels of OVA‐IgE and OVA‐IgG1, while showing increased levels of OVA‐IgG2a (Figure 6C–E). Next, spleen cells were cultured with OVA, and the supernatants were collected to detect Th1‐associated cytokines (IFN‐γ) and Th2‐associated cytokines (IL‐4, IL‐5, and IL‐13) expressions [16]. Splenocytes from these TK‐treated mice demonstrated decreased levels of IL‐4, IL‐5, and IL‐13, along with higher levels of IFN‐γ following OVA stimulation (Figure 7A–D).

### 3.6 TK Treatment Suppressed Cytokine and ROS Production in BEAS‐2B Cells

**FIGURE 8:**
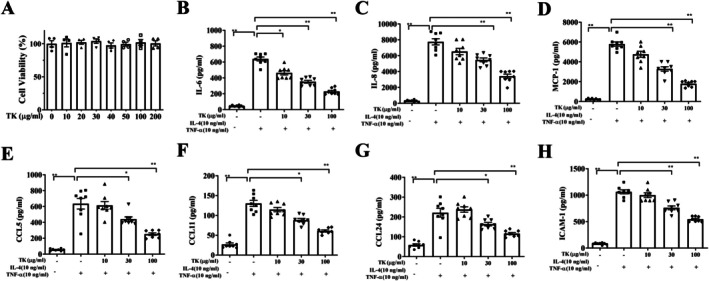
Effects of *T. kirilowii* fruit extract (TK) on cytokine and chemokine production by BEAS‐2B cells. (A) Cell viability of TK‐treated, TNF‐α/IL‐4‐stimulated BEAS‐2B cells. (B–F) ELISA results show the levels of (B) IL‐6, (C) IL‐8, (D) MCP‐1, (E) CCL5, (F) CCL11, (G) CCL24, and (H) ICAM‐1 in BEAS‐2B cells treated with TNF‐α/IL‐4. Data are presented as mean ± SEM. *p < 0.05, **p < 0.01, compared with BEAS‐2B cells stimulated with TNF‐α/IL‐4 without TK pretreatment.

**FIGURE 9:**
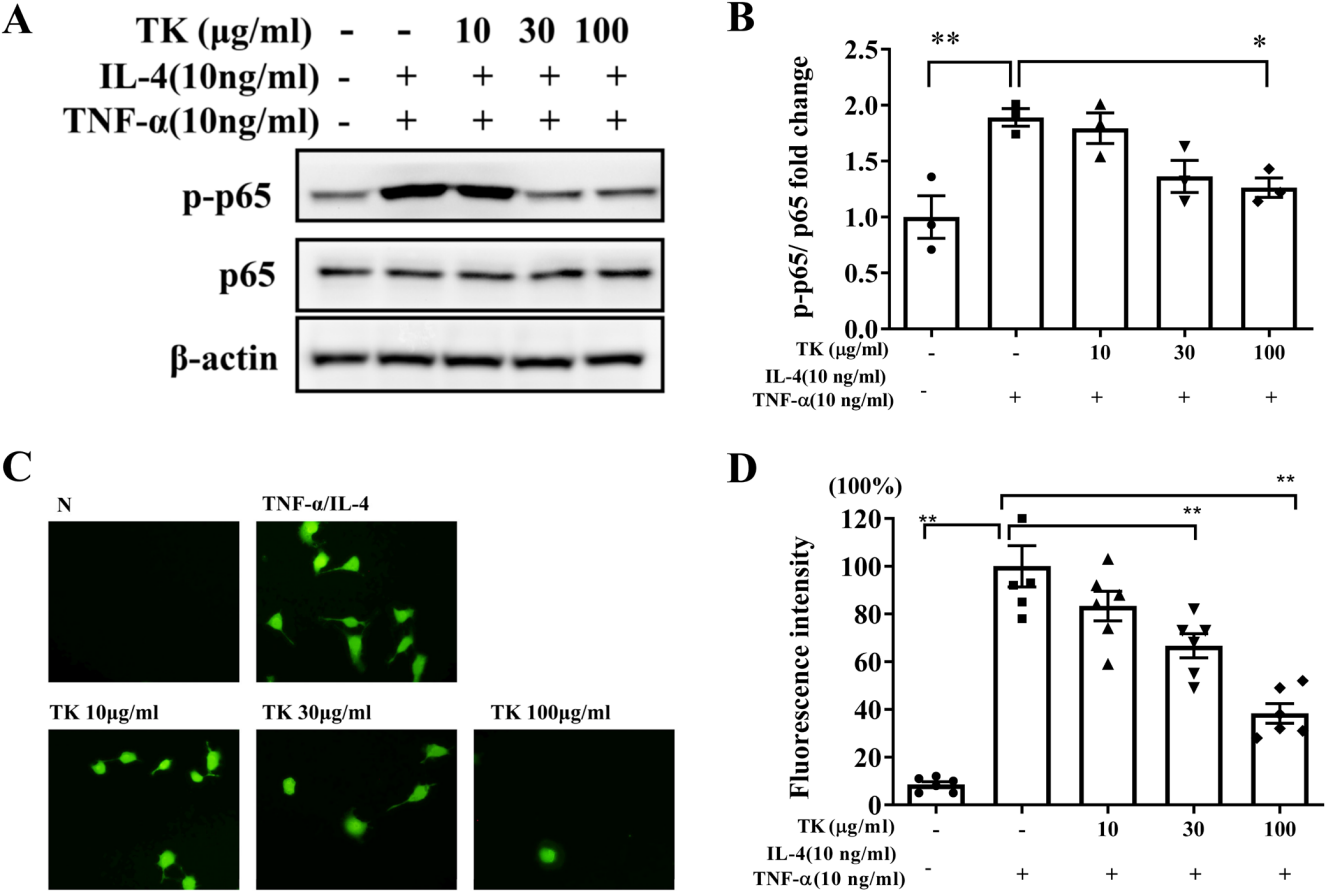
*T. kirilowii* fruit extract (TK) effects on NF‐κB and ROS production in activated BEAS‐2B cells. (A) The phosphorylation of p65 (p‐p65, NF‐κB subunit) expression, and (B) fold‐change relative to p‐p65/p65 expression. (C) Fluorescence microscopy images show ROS labeled with DCF‐DA. (D) Fluorescence‐intensity quantification of ROS production. Values are the mean ± SEM. **p < 0.01 versus TNF‐α /IL‐4 activated BEAS‐2B cells.

The CCK‐8 assay indicated that TK doses ≤ 200 μg/mL had no significant cytotoxic effects on BEAS‐2B cells (Figure 8A). Consequently, subsequent experiments utilized TK doses ranging from 10 to 100 μg/mL. Allergens or proinflammatory cytokines can irritate the inflammatory response and ROS production in bronchial epithelial cells [17]. Inflamed bronchial epithelial cells can release cytokines and chemokines, which aggravate lung inflammation and damage [18]. Pretreatment with TK effectively lowered the levels of IL‐6, IL‐8, CCL5, and MCP‐1 in TNF‐α/IL‐4‐stimulated BEAS‐2B cells compared to untreated cells (Figure 8B–E). Additionally, TK treatment resulted in decreased production of eotaxins (CCL11 and CCL24) and ICAM‐1 (Figure 8F–H). Next, TK treatment significantly suppressed the phosphorylation of p65 (NF‐κB subunit) compared to TNF‐α/IL‐4‐stimulated BEAS‐2B cells (Figure 9A,B). ROS levels were markedly elevated in TNF‐α/IL‐4‐stimulated BEAS‐2B cells compared to unstimulated cells. TK treatment significantly suppressed ROS production in these stimulated cells (Figure 9C,D).

## 4 Discussion

*T. kirilowii* has been utilized in traditional Chinese medicine for centuries [9], with various parts of the plant, such as the fruit, peel, seed, and root, being employed for medicinal purposes. *T. kirilowii* has the ability to remove heat‐phlegm and improve coughs in respiratory diseases [19]. The dried roots of *T. kirilowii* can be used to treat diabetes and improve cough, and can also remove toxins for detumescence [20]. The fruit is known for its ability to treat airway inflammation and reduce phlegm [9]. The South Korean GHX02 and KOTMIN13 formulas (both containing *T*. *kirilowii*) can improve airway inflammation in asthmatic mice [21, 22]. A traditional Chinese medicine formula, NRICM101 (containing *T. kirilowii* fruit), developed by Taiwanese traditional Chinese medicine practitioners, can significantly alleviate respiratory symptoms in patients with COVID‐19 [10]. Previous studies have isolated several purified compounds from *T. kirilowii*, including karounidiol, 3,29‐dibenzoyl karounitriol (DK), and 3,29‐O‐dibenzoyloxykarounidiol [9, 23]. In this study, the HPLC fingerprint analysis of TK identified DK at 16.4 min, confirming its specificity for the identification of *T. kirilowii*. Traditional Chinese medicine often uses complex formulas or a single formula to prevent or treat disease. Our research demonstrated that TK significantly mitigated tracheal goblet cell hyperplasia, collagen accumulation, eosinophil infiltration, and AHR in asthmatic mouse lungs. Furthermore, it reduced inflammation and oxidative stress while decreasing Th2‐related cytokines and chemokines in lung tissues and BALF. TK also inhibited ROS generation and the release of pro‐inflammatory cytokines and chemokines in activated BEAS‐2B cells.

AHR is a critical measure of lung function in asthma patients. The AHR value can mainly be used to detect airway flow to evaluate respiratory system function [3]. Asthmatic patients often experience thickened airway smooth muscles and reduced airway contractility and elasticity, leading to difficulty in breathing during an asthma attack becomes short of breath [24]. Elevated IL‐13 levels in lungs are associated with increased AHR and impaired lung function [25]. IL‐13 secretion by Th2 cells worsens AHR, and excess IL‐13 was measured in lungs and BALF of patients with asthma [16]. IL‐13 knockout mice could markedly reduce AHR in asthmatic mice [26]. Our experiments demonstrate that TK effectively reduces AHR in asthmatic mice, alleviating breathing difficulties and enhancing respiratory function via blocking IL‐13 expression in asthmatic mice.

Allergens can induce tracheal epithelial cells to transform into goblet cells [27], leading to excessive mucus production that obstructs airways during asthma attacks [28]. Without the use of bronchodilators, this condition can lead to severe breathing difficulties or suffocation. Cytokines such as IL‐13 and IL‐4 stimulate goblet cell differentiation and hyperplasia, resulting in excessive mucus secretion [29]. Previous studies showed that IL‐4/IL‐13 could promote sonic hedgehog pathway to cause goblet cell metaplasia and mucous hypersecretion in tracheal epithelial cells [30]. Oxyresveratrol, myricetin and mulberroside F could significantly reduce IL‐4/IL‐13 production in BALF and splenocytes and effectively reduce goblet cell hyperplasia in asthmatic mice [11, 31, 32]. In our present experimental study, we found that TK treatment reduced goblet cell hyperplasia and mucus secretion in asthmatic mice, as confirmed by PAS staining. TK's ability to lower IL‐13 and IL‐4 levels in BALF and spleen cell cultures suggests its potential to mitigate mucus hypersecretion and airway obstruction.

Th2 cell activation leads to increased IL‐5 production, stimulating the differentiation of hematopoietic stem cells into eosinophils [29]. Asthma patients often exhibit high eosinophil infiltration in the lungs and airways. These eosinophils can release more basic proteins and eosinophil cationic proteins that inflame alveolar and tracheal epithelial cells [33], further stimulating mucus production and exacerbating lung damage [28]. Eotaxins (CCL11, CCL24, CCL26) produced by tracheal epithelial cells in response to IL‐4 and TNF‐α attract eosinophils to the lungs [32]. TK treatment effectively suppressed the secretion of CCL11 and CCL24 in inflamed tracheal epithelial cells and reduced ICAM‐1 expression, which blocks immune cell adhesion to tracheal epithelial cells. In asthmatic mice, TK significantly decreased eosinophil numbers in lungs and BALF, mitigating inflammation and oxidative stress. Excess IL‐4 stimulates B cells to produce more IgE, and the interaction of IgE with eosinophils or mast cells in the lungs releases substantial amounts of allergic and inflammatory cytokines, which trigger allergic reactions, inflammation, and oxidative damage [16]. The specific IgG subclass secreted by the B cells depends on the T cell type [15]. Naive T cells can differentiate into two major subtypes, Th1 and Th2 cells. Th1 cells mainly induce cellular immune which promotes the activity of macrophages and CD8 T cells. Th1 cell products IFN‐γ to stimulate B cell populations to secrete IgG2a [16]. Th2 cells primarily promote humoral immunity and can stimulate B cells to secrete antibodies, such as IgG1 and IgE. Therefore, IgG1 and IgG2a expressions can be used as indicators of Th1 cell and Th2 cell activation [4]. Therefore, TK treatment attenuated IL‐4 and IL‐5 expression in BALF and spleen cells, lowered serum OVA‐IgE and OVA‐IgG1 levels (Th2‐related antibodies), and increased OVA‐IgG2a expression (Th1‐related antibody).

Chronic lung inflammation and oxidative stress can lead to collagen accumulation, causing pulmonary fibrosis. This condition stiffens the alveolar cell walls, reduces their elasticity, impairs lung capacity in asthma patients, and hinders efficient gas exchange [34, 35]. Additionally, inflamed immune cells or tracheal epithelial cells release more inflammatory cytokines, further exacerbating alveolar inflammation and promoting collagen deposition in the lungs [3, 18]. The NF‐κB is a transcription factor that can regulate the expression of various pro‐inflammatory genes for the homeostasis of the immune system [13]. TK treatment could attenuate NF‐κB expression in TNF‐α/IL‐4‐stimulated BEAS‐2B cells and the lungs of asthmatic mice. Therefore, TK treatment reduces IL‐6 secretion from inflamed tracheal epithelial cells, as well as IL‐6 and TNF‐α levels in BALF. TK treatment could also attenuate the secretion of MCP‐1, CCL5, and IL‐8 in inflamed epithelial cells, leading to decreased immune cell infiltration, reduced collagen buildup, and improved lung fibrosis.

Inflamed immune cells in the lungs also release ROS, which exacerbate cytokine release and oxidative stress, leading to excessive tracheal smooth muscle contraction and mucus hypersecretion [36]. Excess ROS worsen AHR and cause shortness of breath [37]. TK treatment enhances the production of antioxidant enzymes (SOD, GSH, and CAT) and reduces MDA levels, an oxidative stress marker, improving lung function in asthmatic mice by decreasing ROS levels.

Allergic asthma is mainly caused by patients inhaling allergens such as mites or pollen [38]. Most asthmatic patients have strong Th2 cell activity, which leads to excessive eosinophil infiltration in the lungs [16]. Additionally, cigarette smoke or viral infections may exacerbate asthma symptoms, leading to severe asthma with neutrophil infiltration of the lungs. Neutrophilic asthma may increase the risk of developing steroid resistant [39]. OVA‐induced allergic asthma can effectively promote eosinophil, not neutrophil, infiltration in the lungs of mice [40]. TK‐treated asthmatic mice significantly inhibited eosinophil infiltration in the lungs and reduced the proportion of eosinophils in BALF, which would contribute to reducing eosinophil‐induced inflammation and oxidative damage of the lungs and airways.

TK demonstrates the ability to alleviate airway inflammation and oxidative stress in asthmatic mice. These findings confirm that TK can improve AHR, reduce eosinophil infiltration, and decrease mucus overproduction in asthma, primarily through suppressing Th2 cytokine activity.

## Conflicts of Interest

The authors declare no conflicts of interest.

## Data Availability

The data that support the findings of this study are available from the corresponding author upon reasonable request.
